# Hollow Fiber Membranes of PCL and PCL/Graphene as Scaffolds with Potential to Develop In Vitro Blood—Brain Barrier Models

**DOI:** 10.3390/membranes10080161

**Published:** 2020-07-22

**Authors:** Marián Mantecón-Oria, Nazely Diban, Maria T. Berciano, Maria J. Rivero, Oana David, Miguel Lafarga, Olga Tapia, Ane Urtiaga

**Affiliations:** 1 Department of Chemical and Biomolecular Engineering, ETSIIyT, University of Cantabria, Avda. Los Castros s/n, 39005 Santander, Spain; manteconma@unican.es (M.M.-O.); riveromj@unican.es (M.J.R.); urtiaga@unican.es (A.U.); 2 Instituto de Investigación Marqués de Valdecilla (IDIVAL), Cardenal H. Oria s/n, 39011 Santander, Spain; maria.berciano@unican.es (M.T.B.); miguel.lafarga@unican.es (M.L.); olga.tapia@unican.es (O.T.); 3 Centro de Investigación Biomédica en Red sobre Enfermedades Neurodegenerativas (CIBERNED), 528031 Madrid, Spain; 4 Department of Molecular Biology, University of Cantabria, Cardenal H. Oria s/n, 39011 Santander, Spain; 5 TECNALIA, Basque Research and Technology Alliance (BRTA), Mikeletegi Pasealekua 2, 20009 San Sebastián, Spain; oana.david@tecnalia.com; 6 Department of Anatomy and Cell Biology, University of Cantabria, Cardenal H. Oria s/n, 39011 Santander, Spain; 7 Universidad Europea del Atlántico, Parque Científico y Tecnológico de Cantabria, Isabel Torres 21, 39011 Santander, Spain

**Keywords:** mixed-matrix hollow fibers, graphene, poly(ε-caprolactone), 3D cell cultures, in vitro blood brain barrier (BBB) model

## Abstract

There is a huge interest in developing novel hollow fiber (HF) membranes able to modulate neural differentiation to produce in vitro blood–brain barrier (BBB) models for biomedical and pharmaceutical research, due to the low cell-inductive properties of the polymer HFs used in current BBB models. In this work, poly(ε-caprolactone) (PCL) and composite PCL/graphene (PCL/G) HF membranes were prepared by phase inversion and were characterized in terms of mechanical, electrical, morphological, chemical, and mass transport properties. The presence of graphene in PCL/G membranes enlarged the pore size and the water flux and presented significantly higher electrical conductivity than PCL HFs. A biocompatibility assay showed that PCL/G HFs significantly increased C6 cells adhesion and differentiation towards astrocytes, which may be attributed to their higher electrical conductivity in comparison to PCL HFs. On the other hand, PCL/G membranes produced a cytotoxic effect on the endothelial cell line HUVEC presumably related with a higher production of intracellular reactive oxygen species induced by the nanomaterial in this particular cell line. These results prove the potential of PCL HF membranes to grow endothelial cells and PCL/G HF membranes to differentiate astrocytes, the two characteristic cell types that could develop in vitro BBB models in future 3D co-culture systems.

## 1. Introduction

The application of membranes in the medical field has gained interest in the last few years and represents one of the most relevant markets for membranes. Among the different biomedical applications, the use of membranes for tissue engineering as scaffolds help to ensure sufficient nutrient transport and to promote cell attachment and proliferation mimicking physiological vascular networks and engineering tissues in vitro [1,2,3]. This approach might serve to produce implantable tissue-engineered devices, but also in vitro cell/tissue models that recapitulate tissue functionality to screen new pharmaceuticals and/or to study the mechanisms of the progression of diseases.

With the increasing concern of traumatic brain injuries, neurodegenerative diseases, and brain tumors, the progress of new therapies designed for central nervous system (CNS) illnesses is crucial for ensuring social and economic sustainability in an ageing world. In this line, tissue-engineered models could enable the study of clinically relevant processes such as CNS drug delivery efficiency through the blood–brain barrier (BBB) [4,5]. The BBB is a dynamic and complex structure of brain capillaries that tightly regulates the exchange of nutrients, oxygen, and metabolites between the circulating blood and the extracellular fluids of the nervous tissue in the CNS. The BBB is essential for the control of CNS homeostasis and for the protection of the nervous tissue from toxins or pathogens, and alterations of this barrier underlie the progression of different neurological disorders. However, the BBB represents a critical hurdle in the treatment of CNS diseases as it prevents most pharmaceutical drugs from entering the brain due to its physical, biochemical, and specific barrier properties [5,6]. Interestingly, some neurotransmitters such as dopamine and serotonin involved in the physiopathology of the Parkinson and depression, respectively, cannot cross the BBB; in contrast, some of their precursors can cross this barrier and are used instead to treat these diseases [7]. Therefore, there is a huge interest in the development of reliable in vitro BBB models to help biomedical researchers during the advancement of screening procedures for novel drugs targeted to CNS. Our experimental study may contribute to the design of an in vitro BBB model for testing the permeability of innovative drugs for treating neurological disorders.

A variety of simplified in vitro BBB models have been developed including static, dynamic, stem cell-based, and microfluidic models to understand the dynamics of the BBB [4,5]. Among these, dynamic in vitro BBB models were demonstrated to be a powerful methodology to study the pathophysiology of various CNS disorders since they have the ability to simulate some physiological and pathological properties of the BBB in vivo [8,9,10]. Dynamic in vitro BBB models use artificial capillary supports that usually consist of commercial polypropylene hollow fiber (HF) membrane modules in which endothelial and neural cells are co-cultured. The culture media flows in the intraluminal space at variable speeds, generating shear stress equivalent to in vivo BBB. Besides, these models maintain a stable microenvironment in CO_2_ and O_2_ [4,5].

Nevertheless, the nature and properties of the polypropylene HFs used in the dynamic in vitro BBB existing models reveal a poor and unspecific substrate for cellular attachment and require the application of a suitable matrix molecule (e.g., fibronectin) for successful cell adhesion. Additionally, one of the limitations reported in these studies is that the small average diameter pores of the polypropylene membranes used (0.5 µm) could generate nutrient limitations from the abluminal compartment to the intraluminal space of the HFs and vice versa [9,10]. It is therefore desirable to fabricate a biopolymeric-based material with suitable properties for both the specific differentiation of glial cells and the proliferation of endothelial cells that will mimic some of the BBB barrier properties in vitro.

Nowadays, synthetic biocompatible and bioresorbable polymers are commonly used in constructing tubular structures for tissue regeneration [2,11,12]. Several works have demonstrated that poly(ε-caprolactone) (PCL) membranes can be successfully used as cell culture platforms for bio-hybrid neuronal models, small caliber blood vessel regeneration, and for the development of vascularized human hepatic tissue [13,14,15,16]. Also, a microfluidic perfusion system was recently developed using a blend of PCL and poly(dl-lactide-co-glycolide) (PLGA) made by freeze-coating a 3D-printed sacrificial template [17] as an ex vivo vascularized neural construct. Although the vasculature network of this PCL/PLGA microfluidic perfusion system could be improved to ensure a well-distributed circulatory perfusion, it recapitulated similar in vivo BBB function. Moreover, in the last few years, graphene (G) and derivatives have been explored as interesting nanomaterials for their potential in biomedical applications. In particular, for neural and nerve regeneration, graphene has demonstrated outstanding results either as self-supported scaffolds or as part of a composite material with different polymers [18,19]. Other researchers reported the processability via wet-spinning of liquid crystalline dispersions of graphene oxide to produce microfibers for neuronal interfaces [20,21]. We have previously demonstrated that PCL/graphene-based nanomaterial composites in flat 2D-membrane configuration could be used, even after 1 month, as an optimal and promising cell culture scaffold for neural tissue differentiation [22,23,24]. Furthermore, the study of the hydrolytic degradation of these PCL/graphene-based nanomaterial membranes showed null release of graphene nanoparticles from the polymer matrix during the 1-year period [23]. The PCL/graphene composites incorporate the benefits of PCL, a proven biodegradable and biocompatible material [25] and the ability of graphene to provide electrical conductivity that could modulate the bioelectrical activity of neural cells. So far, few works have fabricated PCL/graphene tubular 3D-scaffolds using solvent evaporation techniques [14] or 3D printing methodologies [26] for tissue engineering applications. In both cases, the electrically conductive 3D graphene scaffolds promoted peripheral nerve regeneration and remyelination after peripheral nerve injury. However, these materials have never been studied for CNS regeneration or as substrates for in vitro BBB models.

Hence, this work aims to explore the potential of PCL and PCL/graphene HF membranes, synthesized for the first time by phase inversion, to be used as 3D cell culture scaffolds. The HFs will be characterized in terms of porous morphology, Raman spectrum, mechanical stability, water transport, and electrical properties. Notably, the cell adhesion, proliferation, and differentiation capabilities of the endothelial and astroglia cells, two cell lines essential for the development of in vitro BBB models, will be analyzed under static conditions.

## 2. Materials and Methods 

### 2.1. Preparation of PCL/Graphene-Based Nanomaterial Hollow Fibers

In the present study, instead of laboratory-made graphene oxide or reduced graphene oxide, which are widely studied in the literature, we decided to explore the feasibility of using commercial graphene/graphite (G) nanoplatelets (Av-PLAT-7, Avanzare) produced at a large industrial scale by mechanical exfoliation. The PCL/G polymer dope solution was prepared as follows: 0.1 wt% of G were homogeneously dispersed by sonication during 20 min in N-methyl pyrrolidone (NMP, 99%, extrapure, Acros Organics, Madrid, Spain). Afterwards, 15 wt% of PCL pellets (MW, 80 kDa, Sigma Aldrich, Madrid, Spain) and 5 wt% of absolute ethanol (EtOH, Sigma Aldrich, Madrid, Spain) were dissolved by rolling (Roller Shaker 6 Basic, IKA, Staufen, Germany). The polymer solution was left to degasify overnight at room temperature. As a control, a PCL dope solution was prepared similarly without graphene.

The PCL and PCL/G HFs were fabricated by non-solvent induced phase separation produced by immersion precipitation using a small dimension spinning set-up to process the polymer solution [27,28]. Polymer solution and bore fluid were simultaneously pumped through a tube-in-orifice spinneret. The formation conditions for the fibers are summarized in Table 1. From each polymer solution, several meters of HF membranes were prepared, and only homogeneous and reproducible sections were selected for experimental purposes.

### 2.2. Physicochemical Characterization of the Hollow Fibers

The structure and morphology of the surface and cross section of the PCL and PCL/G HF membranes were determined using environmental scanning electron microscopy (ESEM, Quanta FEG 250, Thermo Fisher Scientific, Waltham, Massachusetts, USA) with a Large Field Detector (LFD) in low vacuum mode (80 Pa) at a voltage of 5 kV. For the cross-section images, samples were freeze-dried in liquid nitrogen and fractured. The samples were not metal sputtered before examination. The dimensions of the HFs were determined analyzing at least four cross-sectional and outer surface ESEM images taken from sample cut at different lengths of the same HF type (*n* ≥ 4). In addition, the average pore size was estimated using an image processing program (Fiji, ImageJ, U.S. National Institutes of Health, Bethesda, Maryland, USA).

The porosity (ε) of the HFs was quantified using a bulk density method, measuring the mass and dimensions of the membranes, according to Equation (1), where $\rho_{a}$ is the apparent density of the HFs, determined by the calculation of the mass/volume ratio for three samples of each HF type (*n* = 3), and $\rho_{b}$ is the density of PCL polymer (1.145 g cm^−3^). The density,${\;\rho }_{b}$, of PCL/G composite was estimated as 1.139 g cm^−3^ [29].

(1) $$\varepsilon =\left(1-\frac{\rho_{a}}{\rho_{b}}\right)\cdot 100\;\left(\%\right)$$

The viscosity of the polymeric solutions was measured using a rotational viscometer (Fungilab, Alpha Series, Barcelona, Spain) utilizing a TL5 spindle with rotation speeds of 0.5–0.6 rpm and at room temperature.

The PCL and PCL/G HFs were analyzed by Raman spectroscopy. The analysis was carried out using InVia Reflex (Renishaw, Gavá, Spain) at the Surface Analysis Center of Tecnalia (Donostia, Spain). A 785 nm wavelength beam from a krypton–argon ion laser was focused with a 50x objective for detection. The analysis was performed from 100–3200 cm^−1^ at 50% power with an exposition time of 10 s and 10 accumulations.

### 2.3. Mechanical, Electrical and Flux Properties Characterization of the Hollow Fibers

Axial tensile tests of the HFs were done using specimens of 50 mm active length (MECMESIN mod. MultiTest 5-i n/s, 17-1109-03) with a load cell capacity of 100 N at a constant speed of elongation of 50 mm min^−1^. The average values for the strain properties were obtained from five samples of each fiber (*n* = 5).

The electrical conductivity of the PCL and PCL/G HF dry membranes was evaluated by electrical impedance measurements with a PM 6304 programmable automatic RCL meter (Philips, Eindhoven, The Netherlands). Alligator clips were placed at a distance of 0.5 cm along the length of the HFs. The tests were carried out for at least three samples of each HF type (*n* ≥ 3) at room temperature using a frequency of 100 Hz (attending to the routine frequency range used in electroencephalography for clinical diagnosis and for treatment in CNS between 0.5 and 100 Hz [30]). The equipment provided with the values of impedance module (*Z*) and phase angle (*φ*). The electrical conductivity calculations for the HFs are described elsewhere [24].

The hydraulic permeability was determined using a dead-end flow filtration system at room temperature. The feed reservoir (800 mL, Amicon^®^, Millipore, Madrid, Spain) contained ultrapure water that was pressurized with air in the HF membrane module. Each membrane module contained two HFs of 13.3 cm length for PCL and 24.2 cm for PCL/G with a total effective membrane area, *A_e_*, of 9.8 and 21.4 cm^2^, respectively. The permeate side was opened to the atmosphere. The volume of water that permeated through the HFs was continuously collected and its weight was automatically recorded in real time with Pomiar Win software (*W_w, permeated_*). The water flux, *J_w_* (L m^2^ h^−1^), was measured at fixed pressure values during up-down pressure cycles, Δ*P*, within 0.4–1.5 bar and was determined using Equation (2), where Δ*t* (h) is the time period of permeate collection. The hydraulic permeance, *P_w_* (L m^−2^ h^−1^ bar^−1^), was calculated according to Equation (3).

(2) $$J_{w}=\frac{W_{w,permeated}}{∆t\;\cdot {\;A}_{e}\;\cdot {\;\rho }_{w,\;22\;{}^{{}^{\circ}}C}}$$

(3) $$P_{w}=\frac{J_{w}}{∆P}$$

Prior to the filtration tests, the HFs were immersed in a 70% *v*/*v* EtOH solution for 30 min simulating the sterilization method. The hydraulic permeation tests were conducted at least in duplicate for each membrane (*n* ≥ 2).

The thickness of the selective layer (δ) on the outer surface of the hollow fibers was estimated assuming the Hagen–Poiseuille porous model. In Equation (4), $d_{p}$ refers to the effective mean surface pore diameter that was evaluated from ESEM images, μ is the dynamic viscosity of ultrapure water (9.55 × 10^−4^ kg m^−1^ s^−1^) through the membrane at 22 °C, and τ refers to the tortuosity of the material that was calculated using Equation (5) [31].

(4) $$\delta =\frac{\varepsilon \;\cdot {\;d}_{p}^{2}}{32\;\cdot {\;P}_{w}\;\cdot \;\mu \;\cdot \;\tau }$$

(5) τ=1−0.49·ln(ε)

### 2.4. Cell Cultures

For the cell culture experiments, samples of the PCL and PCL/G HFs were cut into 0.5 cm length and placed on flat bottom 6-well plates (CorningTM). The experimental configuration consisted of two samples of each HFs (PCL and PCL/G) and two 10 mm diameter glass coverslips (Tissue Culture Plastic, TCP) as control in each well plate. Each sample was sterilized with 70% v/v EtOH in distilled water for 30 min and subsequent exposure to UV light for 15 min in a laminar cabinet. After sterilization, the HFs were washed three times with phosphate-buffered saline (PBS) to remove ethanol traces.

Rat C6 glioma cells (ATCC^®^ CCL-107^™^) were maintained in Dulbecco’s Modified Eagle Media (DMEM, Gibco^™^, Thermo Fisher Scientific, Waltham, Massachusetts, USA) with 4.0 mM L-Glutamine supplemented with 10% fetal bovine serum (FBS, Gibco^™^), 1% of antibiotic agents (penicillin G and streptomycin), and 1% of nonessential amino acids. For the differentiation of C6 cells towards astrocytes, 1 mM of dibutyryl cAMP (dbcAMP, Sigma Aldrich, Madrid, Spain) and 0.25% of FBS were added to the cell culture media. This treatment inhibits cell proliferation and changes the phenotype of C6 cells to astrocytes [32]. Human umbilical vein endothelial cells (HUVECs) (ATCC^®^ CRL-1730™) were maintained in Vascular Cell Basal Medium (VCBM) (ATCC) supplemented with Endothelial Cell Growth Kit-BBE (ATCC) which consisted of 2% FBS, 0.2% bovine brain extract, 5 ng mL^−1^ rhEGF, 10 mM L-glutamine, 0.75 U mL^−1^ heparin sulfate, 1 μg mL^−1^ hydrocortisone hemisuccinate, 50 μg mL^−1^ ascorbic acid, penicillin (20 U mL^−1^), and streptomycin (20 μg mL^−1^) (Sigma Aldrich, Madrid, Spain) at 37 °C in a humid atmosphere containing 5% CO_2_.

For the cell adhesion experiments, cells were trypsinized from culture flasks and resuspended in culture medium. A cell suspension containing 3 × 10^5^ cells was added to each well and the cells were led to seed by gravity onto the HFs and TCP controls. Cell culture plates were kept at 37 °C in a humid atmosphere containing 5% CO_2_.

#### Immunofluorescence and Confocal Microscopy

Cells were fixed with freshly prepared paraformaldehyde (3.7% in PBS) and permeabilized with Triton X-100 (0.5% in PBS) for 15 min at room temperature. Phalloidin-FITC conjugate (Sigma Aldrich, Madrid, Spain), which binds polymerized F-actin, was used to identify cytoskeletal actin filaments, while DAPI (4′, 6-diamidino-2-phenylindole) was used to mark the DNA and to recognize cell nuclei. All samples were mounted with antifade medium Vectashield (VectorLabs, Barcelona, Spain) and were examined with a Nikon A1R confocal scanning laser microscope. For the analysis of cells grown on the HFs, the samples were inverted and a series of optical sections were obtained using the objective Plan Apo 20X DIC N2 equipped with a 405 nm argon laser and 561 nm HeNe laser to detect cell nuclei (DAPI) and cytoskeleton (Phalloidin-FITC), respectively. The reconstructions of 37 images (15 μm step^−1^) of confocal sections were assembled using NIS Elements 3.2 software (Boston, Massachusetts, USA).

### 2.5. Statistical Analysis

Quantitative cellular experiments were run in duplicate for each cell line and substrate. For the adhesion, proliferation, and differentiation cellular stages, the total number of cells was counted from 4 micrographs and expressed per square millimeter. To evaluate the differentiation stage of the C6 cell line a morphometric analysis was done to measure the length of the processes, defined as the extension from the point where the process emerged from the cell body to the tip of the major process. The number of mature astrocytes, defined as cells with at least two processes longer than the cell body, were counted from each image and the percentage of differentiated cells was calculated. 

All data were presented as mean ± standard deviation (mean ± SD). Statistical analyses were performed using GraphPad Prism 7 (GraphPad Software, La Jolla, CA, USA). Statistical differences between two groups were assessed by using paired two-sided Student’s t-test. Statistical differences among multiple groups were evaluated by one-way analysis of variance (ANOVA) followed by Bonferroni correction post-hoc tests. The statistical significance level was defined at *p* < 0.05.

## 3. Results and Discussion

### 3.1. Physicochemical Characterization of the Hollow Fibers

**Figure 1:**
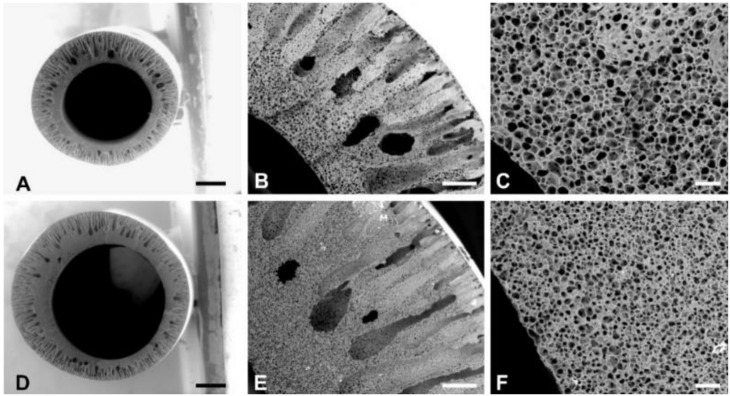
Cross-section ESEM images of (**A**–**C**) PCL and (**D**–**F**) PCL/G HFs employed to determine the HFs’ morphological characteristics as shown in Table 2. (**B**,**C**) and (**E**,**F**) are image magnifications of (**A**) and (**D**), respectively. Scale bar: 250 µm (**A**,**D**), 50 µm (**B**,**E**) and 10 µm (**C**,**F**).

**Figure 2:**
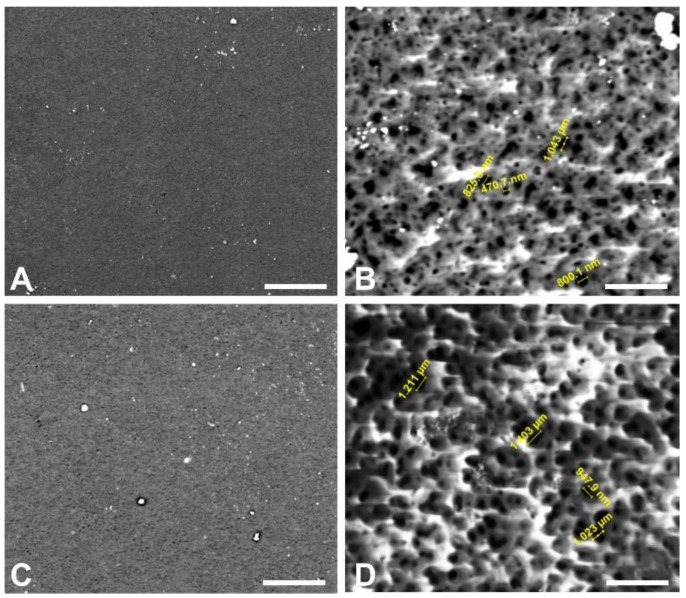
Outer surface ESEM images of the (**A**,**B**) PCL and (**C**,**D**) PCL/G HFs. (**B**,**D**) show the higher magnification details of the external surface of PCL and PCL/G HFs, respectively. A selection of several images of the external surface at high magnification were employed to determine the average external pore diameters using Fiji (Image J) (numbers in yellow). Scale bar: 50 µm (**A**,**C**) and 5 µm (**B**,**D**).

Figure 1 and Figure 2 show representative ESEM images of the cross-section and the outer surface, respectively, of the PCL and PCL/G HFs. From the cross section of PCL (Figure 1A) and PCL/G (Figure 1D), HFs with a porous morphology were observed. At a higher magnification, the porous structure of the cross-section of both HFs was asymmetric and contained finger-like cavities (macro-voids) reaching from two third of the wall towards the outer surface, and sponge-like structure with micropores towards the internal diameter (Figure 1B,C,E,F). The external macrovoid structure of the cross section of the PCL and PCL/G HFs was formed due to instantaneous demixing during the phase inversion, via nucleation produced by the high affinity of the coagulant contained in the coagulation bath (90 wt% water, Table 1) towards the solvent of the polymer solution (NMP) [33]. For its part, the sponge-like morphology of the internal pores was the consequence of more retarded coagulation kinetics due to the high content of NMP (80 wt%) in the bore solution co-extruded with the polymer solution. Interestingly, the interconnectivity of the pores observed in the membranes could potentially facilitate nutrients supply and metabolites removal and benefit the molecular transfer between co-cultured cells.

The ESEM images of the outer surface of PCL (Figure 2A) and PCL/G (Figure 2C) HFs showed uniform surface morphology. At a higher magnification (Figure 2B,D) the values of mean surface pore size could be estimated (Table 2). These were significantly smaller for PCL HFs than for PCL/G HFs (0.71 ± 0.04 µm and 0.89 ± 0.08 µm, respectively, Table 2). The increase in the average pore size compared with polypropylene commercial HFs used in BBB models (0.5 µm) [9,10], could be beneficial, as the PCL and PCL/G HFs would allow a higher diffusion of nutrients and mass transfer to the cells. Several studies have reported that the loading of different fillers such as carbon-based nanomaterials in diverse polymer solutions affected the pore size of the composite membranes [34,35]. These works also observed that there was a link between the surface pore size and the viscosity of the dope solution. Although the increase of dope solution viscosity should result in a decrease of solvent–coagulant exchange and smaller surface pores size, remarkably, it was found that the threshold concentration of nanoparticles below which the surface pore size of the composite membranes increased progressively with the viscosity of the polymer solution. In the present study, the dope solution viscosity slightly increased from 1950 ± 37 cP to 2837 ± 79 cP for PCL and PCL/G HFs, respectively. The low concentrations of nanoparticles used in this case (0.1 wt% graphene) may not overcome the aforementioned concentration threshold and could also actuate as nucleating agents or crosslinking points with the polymer chain accelerating the diffusion of the solvent from the polymer solution to the non-solvent and creating bigger surface pores size by thermodynamic instability [34,36]. The higher viscosity of PCL/G dope solution with respect to PCL dope solution may have produced a higher shear stress in the extrusion needle causing the expansion of the internal and external diameters of the PCL/G HFs (see Table 2).

Bulk porosity in both HFs was above 80%, similar to other polymer scaffolds manufactured by phase separation [37], which guaranteed the uniform supply of nutrients to cultured cells. Table 2 summarizes the results obtained from the morphological characterization and porosity of the HFs.

**Figure 3:**
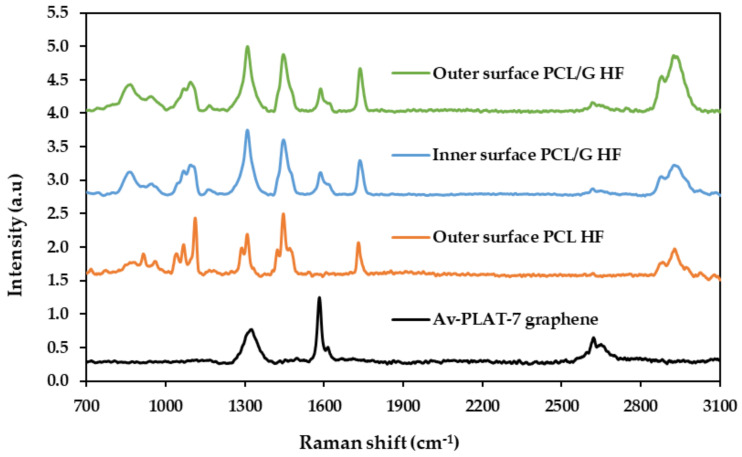
Raman spectra of PCL outer surface (orange), PCL/G inner surface (blue) and PCL/G outer surface (green) HFs and the graphene nanomaterial powder (black) excited with a 785 nm laser showing the characteristic peaks analyzed for the polymer (PCL), the nanomaterial (G), and the composite HFs.

Figure 3 shows Raman spectra of the PCL and PCL/G HFs and graphene nanomaterial. Four relevant peaks in the spectra of the commercial graphene were identified: the D band at 1325 cm^−1^ associated with the presence of carbon atoms with sp^3^ hybridization or structural disorders in the graphite network, the G band at 1583 cm^−1^ attributed to the energy of sp^2^ bonds of C atoms, the D´ band at 1615 cm^−1^ superimposed with the G band and related with edge defects, and, finally, the 2D band at 2635 cm^−1^ associated with the multilayered character of the graphene [38,39]. The I_2D_/I_G_ ratio was 0.35 indicating that the nanomaterial had 6–7 layers [38,40]. 

Regarding the Raman spectrum of pure PCL HFs, the peaks around 922 and 1071 cm^−1^ were attributed to the stretching vibrations of the polymer matrix and the C-C chemical bonds (C-COO group). On the other hand, the peaks at 1300–1330 cm^−1^, 1415–1475 cm^−1^, and 2937 cm^−1^ represented the vibrations of the characteristic CH_2_ bonds of the PCL polymer structure by bending and stretching while the peak at 1735 cm^−1^ corresponded to the C=O bond of the polymer [41]. The inner surface of the PCL HF showed the same pattern.

For the composite scaffolds (PCL/G), the spectra presented the characteristic peaks of PCL and the D, G, D’, and 2D bands of graphene. Furthermore, the intensity and shape of the characteristic peaks were similar in the internal and external surface of PCL/G HFs confirming the homogeneous distribution of graphene in the polymer matrix. The displacement of the 2D and D bands from 2635 cm^−1^ to 2617 cm^−1^ and from 1325 cm^−1^ to 1310 cm^−1^, respectively, might be due to residual tensile stress between the polymer chain and the nanomaterial.

### 3.2. Mechanical, Electrical and Flux Properties Characterization of the Hollow Fibers

The mechanical stability of the polymer scaffolds is important to allow cell adhesion and proliferation [42]. Moreover, the mechanical properties of the HFs must maintain the structure and integrity to ensure the growing and maturation of cultured tissues. Table 3 collects the Young modulus, yield point, ultimate tensile strength, and elongation at break obtained from the axial tensile tests of PCL and PCL/G HFs.

The mechanical properties, particularly elastic modulus, play an important role in cell fate and differentiation. As shown in Table 3, the presence of graphene in the polymer matrix reduces the mechanical properties of the HFs (Young Modulus, ultimate tensile strength, and elongation at break). This reduction from plain PCL HFs to PCL/G HFs was consistent with previous observations on PCL flat membranes doped with reduced graphene oxide (rGO) nanomaterials [22], where graphene nanomaterials restricted the continuity and mobility of PCL chains resulting in the brittleness of the polymer membranes that incorporated nanoparticles [43]. This also confirms that graphene may act as a nucleating agent of the polymer during the phase inversion process. The same tendency in the mechanical parameters was observed for covalently linked graphene/polycaprolactone composites with low loadings of graphene (0.1 wt%) [44]. Likewise, Diban et al. [15] and others [16,45] had reported a similar range of the Young Modulus (14–20 MPa), ultimate tensile strength (1.4–2.6 MPa), and elongation at break (300−522%) for PCL HFs or flat PCL membranes fabricated by phase inversion. Overall, the mechanical properties of the PCL and PCL/G HFs that were prepared in this work should be sufficient to withstand repeated cycles of compression and relaxation as the systole/diastole pressures typical of in vivo blood vessels [27]. Although the PCL and PCL/G HFs yet importantly mismatch the mechanical properties of native biological tissue (i.e., <1 kPa [46]), PCL, as a soft material, present much closer Young Modulus values to native tissues than current commercial PP HF membranes (i.e., 124 MPa for PP HFs manufactured by Phase Separation [47]) used as the benchmark for dynamic in vitro BBB models. Furthermore, a compromise between mechanical biomimetics and structural integrity to sustain pressure during perfusion must be attained.

The electrical conductivity of the HFs was calculated by the electrical impedance measurements at 100 Hz. PCL/G HFs possess significantly enhanced (1.9-fold increase) electrical conductivity (1.42·10^−4^ ± 1.05·10^−5^ S m^−1^) compared to PCL HFs (7.54·10^−5^ ± 2.64·10^−6^ S m^−1^). Such electroactive properties could be critical for electrically excitable tissues to facilitate cell-to-cell communication [48]. Several reports have shown that the incorporation of graphene-based nanomaterials improved the electrical conductivity of the scaffolds [48,49]. The differences in the electrical properties of the membranes could be attributed to the high recognized conductivity of graphene in comparison to PCL polymer that has the typical behavior reported for electrical insulators [18,42].

**Figure 4:**
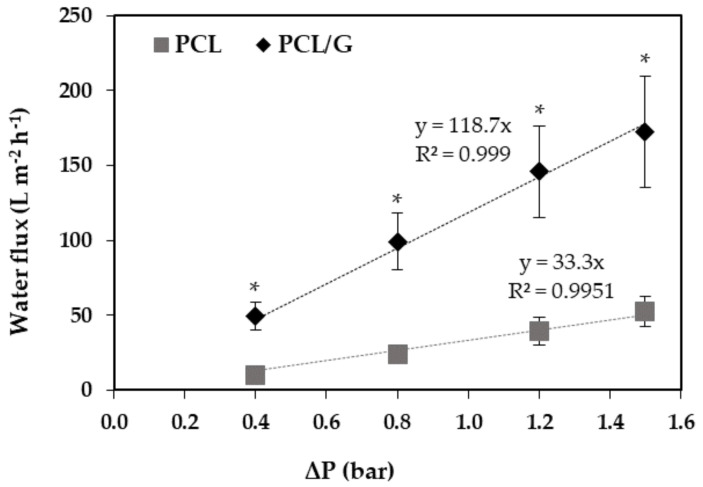
Ultrapure water flux at different transmembrane pressures across the PCL (grey) and PCL/G (black) HFs. The bars represent the mean ± SD. Student’s t-test statistical analysis of significance considering PCL HF as reference (*n* ≥ 2), * *p* < 0.05.

Figure 4 illustrates ultrapure water fluxes (L m^−2^ h^−1^) for different transmembrane pressures (bar).

Overall, the water permeance results were consistent with the ESEM imaging, which showed high porosity values of the HFs. The much lower membrane permeance of the PCL HFs of the present work (33 ± 7 L m^−2^ h^−1^ bar^−1^) in contrast to the PCL HFs reported by Salerno et al. [16] (238 L m^−2^ h^−1^ bar^−1^) is in agreement with the higher external surface pore size (1.3 µm [16] vs. 0.71 µm (current work)) and pore morphology of the PCL HFs prepared in that work. However, in the present study, the PCL/G HFs presented significantly higher hydraulic permeances 119 ± 13 L m^−2^ h^−1^ bar^−1^, than PCL HFs. This improved performance was attributed to the lower mean thickness of the selective layer and the larger pore size at the surface observed in the PCL/G compared to PCL HFs (Table 2). Such high values of permeance may be convenient for application in perfusion systems to provide effective nutrient transport to the cells and to facilitate excreted molecule diffusion. The PCL HFs’ permeance values could be comparable to PLGA and polyetheretherketone (PEEK-WC) HFs, while PCL/G HFs to polylactic acid (PLA) and polyacrylonitrile (PAN) HFs (Table 4). In particular, PAN hollow fiber membranes showed sufficient permeability (146 L m^−2^ h^−1^ bar^−1^) for the correct growth and differentiation of SHSY5Y neuroblastoma cells and their use as neuronal models [50].

### 3.3. Cell Cultures Viability on the Hollow Fibers

In order to test whether the PCL and PCL/graphene HFs could be used as scaffold for cell cultures and be suitable for producing in vitro BBB models, the morphology and the adhesion, proliferation, and differentiation capacity of C6 (astrocyte model) and HUVEC (endothelial model) cells were analyzed on both membranes. The results obtained were compared with TCP as positive control.

**Figure 5:**
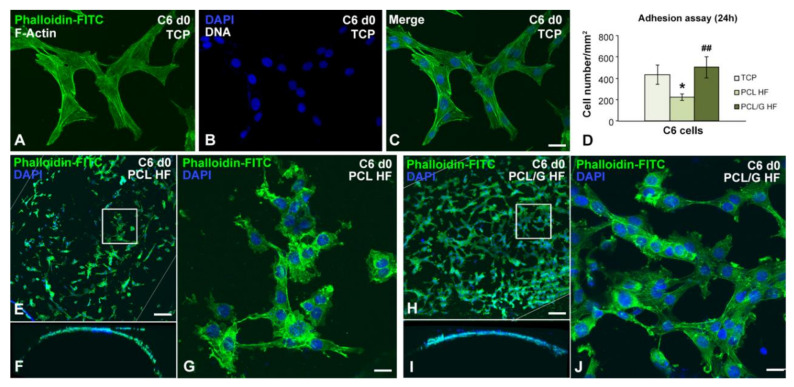
Representative images of confocal microscopy where the density and cell morphology of C6 cells were observed 24 h after sowing (day 0) on (**A**–**C**) TCP, (**E**–**G**) PCL, and (**H**–**J**) PCL/G substrates. At this time, 1 mM dbcAMP was added to the culture medium to induce astrocyte differentiation for 72 h. Cytochemical techniques have been used with Phalloidin-FITC (green), direct marker of the actin cytoskeleton, and DAPI (blue), for the cell nucleus. (**D**) Quantification of the adhesion assay of C6 cells on the indicated substrates after 24 h. *p = 0.0317 versus TCP; ^##^*p* = 0.0058 versus PCL HF (*n* = 4 per group; one-way ANOVA with Bonferroni correction). Scale bar: 20 µm (**A**–**C**,**G**,**J**), 75 µm (**E**,**F**,**H**,**I**).

In the control TCP cultures, undifferentiated C6 cells exhibited a fusiform morphology with numerous F-Actin positive stress fibers (Figure 5A–C). Noteworthy, the microscopic observation of C6 cells grown on PCL HFs showed the loss of their characteristic fusiform morphology, with a more irregular shape and an apparent disappearance of the stress fibers (Figure 5G). In contrast, C6 grown on PCL/G HFs preserved the morphology of the C6 grown on TCP (Figure 5C,J), indicating that the presence of graphene in the HFs allowed them to maintain their normal cellular morphology.

Regarding the adhesion ability of C6 cells after 24 h of seeding, the quantitative analysis of the number of cells per unit area revealed a significant decrease in C6 cell density in PCL HFs compared to TCP, while the cell adhesion on PCL/G HFs showed no significant differences to TCP, suggesting that the functionalization of PCL HF with graphene improves C6 cell adhesion (ANOVA, *p* < 0.005; Figure 5D). Nevertheless, it is important to note that the HF adhesion was quantified considering the whole external surface of the HF while, due to the gravity seeding procedure used in the present study, cells only could deposit on the top part of the surface of the HF.

Additionally, it is well known that PCL is a hydrophobic material with low cell adhesion capacity [17]. It is therefore a common practice to coat the PCL substrates with extracellular fibers such as collagen of fibronectin or hydrogels to favor cell adhesion (both for endothelial or neural types) [17] or to condition the surface with medium containing serum proteins [16]. In the present study, the HF surfaces were not pretreated or coated that could mask plain cell–substrate interactions during adhesion. These results indicate that the simple presence of graphene on the PCL matrix enhanced the adhesion ability of the material without the need of precoating the surface. This enhancement could be associated with the high capacity of graphene to upregulate the expression of vinculin to promote a high number of focal adhesions [54].

**Figure 6:**
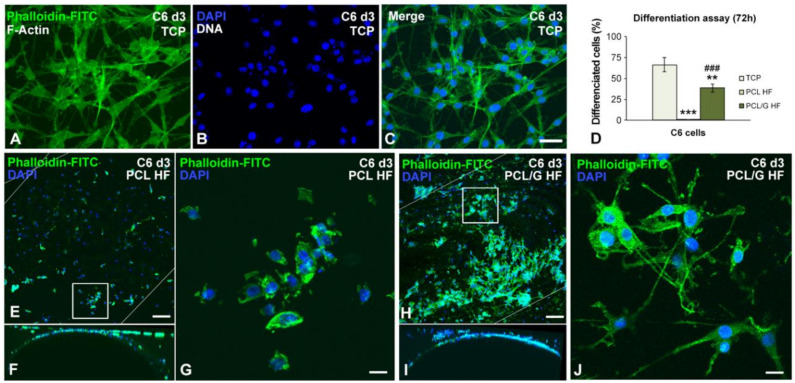
Representative images of confocal microscopy for the study of astrocyte differentiation for 72 h (day 3) on (**A**–**C**) TCP, (**E**–**G**) PCL and (**H**–**J**) PCL/G substrates. In (**C**,**J**), cells had dramatically reduced their cell and nuclear size in addition to presenting numerous cytoplasmic projections. (**D**) Quantification of the differentiation assay of C6 cells on the indicated substrates after 72 h of the adhesion stage. ** *p* = 0.0007, *** *p* = 5.71 × 10^−7^ versus TCP; ^###^ *p* = 5.55 × 10^−58^ versus PCL HF (*n* = 4 per group; one-way ANOVA with Bonferroni correction). Scale bar: 20 µm (**A**–**C**,**G**,**J**), 75 µm (**E**,**F**,**H**,**I**).

To evaluate the differentiation capacity of C6 cells to a glial phenotype, cell cultures were treated with 1 mM dbcAMP for 72 h [32]. The differentiated C6 cells grown in control TCP exhibited dramatic morphological changes including the formation of several long processes and the reduction of their nuclear size (Figure 6A–C). The microscopic analysis on PCL HFs, showed that C6 cells exhibit a great reduction in the Phalloidin-FITC staining of the actin cytoskeleton and no cytoplasmic process were observed (Figure 6E–G). The immunostaining of GFAP on control flat PCL membranes (Supplementary Figure S1) revealed that after day 2 of differentiation, PCL flat membranes showed a limited percentage of C6 cells expressing GFAP and their morphology resembled to that observed in PCL HF membranes. On the contrary, C6 cells grown on PCL/G HFs exhibited a star-shaped astrocyte morphology with long cellular processes (Figure 6H–J). Noteworthy, from the lateral view of the PCL/G fibers, cell migration was also observed (Figure 6I). The quantitative analysis of the differentiation assay revealed that a high proportion of C6 cells were differentiated into astrocytes when grown in PCL/G HFs (38.5 ± 4.8%), while C6 failed to differentiate in PCL HFs (ANOVA, *p* < 0.0005; Figure 6D). PCL/graphene-based flat membranes containing graphene oxide (GO) or reduced graphene oxide (rGO) (Supplementary Figure S1) presented a significantly higher GFAP expression and the morphology as the differentiated C6 on the PCL/G HF surface. This result shows that the potential use of graphene industrially produced by mechanical exfoliation as an alternative to lab-made graphene through Hummer’s method embedded on a biocompatible polymer matrix for biomedical applications, at least at the low concentrations used in the present study. Furthermore, Supplementary Figure S2 reveals negligible fluorescent background which is of utmost importance to conduct (without interferences) future fluorescent assays, i.e., calcium imaging.

The outcome obtained in the C6 cellular model of astrocytic differentiation is in agreement with previous work [24,55]. Thus, using cultures of neural progenitor cells (NPCs), Sánchez-González et al. [24] demonstrated that PCL-graphene flat membranes improved neural cell modulation as compared to PCL membranes. Rastogi et al. [55] evidenced that pristine graphene promotes cell adhesion and proliferation of both nonneuronal (COS-7, fibroblast) and neuronal cells. In this context, it is well established that sp^2^-hybridized carbon atoms, ionic bonds, and hydrophobic interaction between molecules allow graphene-based composite to be conductive and absorb proteins which is important for enhancing biocompatibility, cell growth, and proliferation [18,42]. Our results suggest that the higher conductivity of PCL/G HFs could promote the growth of cytoplasmatic extensions and serve as a guidance for C6 migration and differentiation.

**Figure 7:**
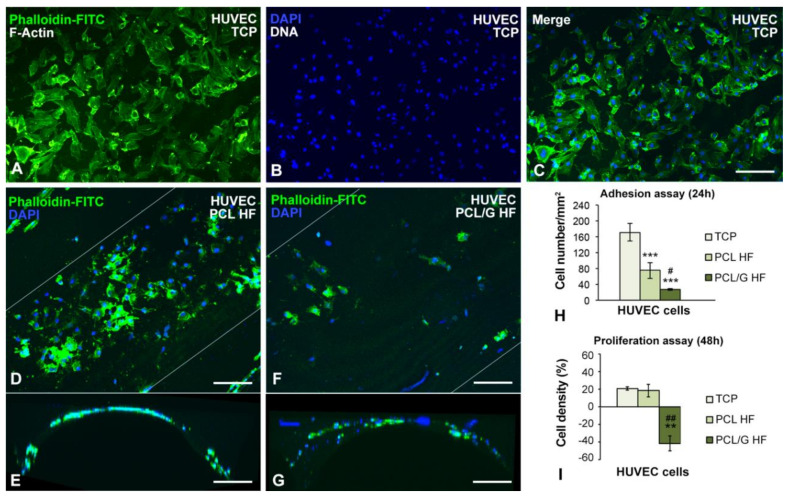
(**A**–**C**) Mosaic cell culture of HUVEC endothelial model over TCP. Confocal microscopy images of HUVEC cells grown on the surface of (**D**) PCL or (**F**) PCL/G membranes in hollow fiber configuration 24 h after sowing (adhesion assay). Side view of the fibers of (**E**) PCL and (**G**) PCL/G. (**H**) Quantification of the cell number per mm^2^ in the adhesion stage (24 h). *** *P* = 0.0002 PCL HF, *** *P* = 8.68 × 10^−6^ PCL/G HF versus TCP; ^#^ *p* = 0.0249 versus PCL HF (*n* = 4 per group; one-way ANOVA with Bonferroni correction). (**I**) Cell proliferation assay where is expressed, in percentage, the increase in the number of cells per mm^2^ after 48 h of the adhesion stage. ** *p* = 0.0018 PCL/G HF versus TCP; ^##^ *p* = 0.002 versus PCL HF (*n* = 4 per group; one-way ANOVA with Bonferroni correction). Scale bar: 100 µm (**A**–**C**), 200 µm (**D**–**G**).

Next, we evaluated the HUVEC endothelial cell model, an important component of the BBB, determining the differential impact of PCL and PCL/G substrates on their adhesion and proliferation capacity. Thus, HUVEC cells were cultured until they reached confluence (24 h) and were kept in culture for an additional 48 h. HUVEC cells tightly associate in a mosaic of polygonal and flattened cells after 24 h of seeding in control TCP cultures (Figure 7A–C). In the case of HUVEC cells grown on HFs, they were arranged in monolayer on the outer surface of the PCL HF membranes with lower cell density than observed in control TCP cultures (Figure 7C–E). Moreover, cell density was dramatically reduced in PCL/G HFs, compared to the other substrates (Figure 7A,D–G). The quantitative analysis confirmed that the number of cells per mm^2^ was significantly lower in PCL/G HFs (< 20%) than in PCL HFs (< 50%) when both were compared to TCP (100%) (ANOVA, *p* < 0.0005; Figure 7H). This reduction in the number of attached cells in HFs respect to TCP could be attributed to factors related to cell seeding on 3D tubular structures that relies on cell contact and subsequent adhesion on a curved surface. Besides, the hydrophobicity of PCL material may also be responsible for the lower HUVEC cell adhesion than in TCP. Furthermore, the cell density for each substrate, expressed in a percentage normalized to 100% at 24 h, increased by 20.7 ± 1.7% and 18.5 ± 7.2% at 48 h on TCP and PCL HFs substrates, respectively (ANOVA, *p* < 0.005; Figure 7I), the difference not being statically significant. This finding reflects the existence of cell proliferation on the PCL substrate. In contrast, a negative growth with reduced cell density was found when HUVEC cells grown on PCL/G HFs (Figure 7I).

It is noteworthy that HUVEC grew on PCL HFs at a normal ratio (Figure 7I) and no apoptotic cells were detected, indicating that PCL HFs are adequate scaffolds for the establishment of an in vitro endothelial cell model. These results agree with the positive biocompatibility of endothelial cells on PCL HFs reported by Salerno et al. [16]. Previous studies using human adipose stem cells in an in vitro model of vascular tissue regeneration also reported a positive cell adhesion and proliferation on PCL HFs [15,27], similar to that observed in the present work. Nevertheless, the incorporation of graphene in the polymeric matrix of PCL/G HFs seemed to induce a negative effect on HUVEC cells. Several studies have shown the biocompatibility and cytotoxicity of HUVEC cell lines exposed to dispersions of graphene-based nanomaterials [56,57]. The mechanistic toxicity studies of Sasidharan et al. [56] revealed that the interaction of few-layer graphene with HUVEC cells induces oxidative stress with mitochondrial superoxide generation, lipid peroxidation, and mitochondrial membrane depolarization, all leading to cell death by apoptosis/necrosis. Moreover, Das et al. [57] reported that graphene oxide and rGO induced a significant increase in both reactive oxygen species (ROS) generation and the expression of mRNAs encoding heme oxygenase 1 (HO1) and thioredoxin reductase (TrxR) in HUVEC cells. However, in our previous work [23], we demonstrated that rGO nanoplatelets remained immobilized on the polymeric matrix even after 1 year of hydrolytic degradation. Therefore, a potential toxic effect caused by G nanoplatelets leaching seems implausible [58]. On the other hand, proteins typically adsorb on hydrophobic graphene substrates through van der Waals and π-π staking of aromatic rings of proteins on the graphene basal plane [59]. Particularly, Lee et al. [60] found that strong π-π interactions between phenyl rings of insulin and CVD-grown graphene surface resulted on secondary and tertiary structural changes of insulin and the consequent impediment to conduct adipogenic differentiation. Insulin has been found to also act as an important regulator for the proliferation and viability of HUVEC cells [61]. Although the mechanism affecting the present situation is not clear yet, taken together, these studies support the hypothesis that the presence of graphene/graphite nanoplatelets in the polymeric matrix negatively affect the proliferation and viability of HUVEC cells.

In summary, this report reveals that PCL/G composite HF is a permissive scaffold that allows C6 cells’ growth, migration, and correct differentiation process into astrocytes. The PCL/G HFs exhibit excellent properties such as electrical conductivity and biocompatibility, making them good candidates for studies of astrocyte cell models. However, the potential toxicity of graphene observed for HUVEC cells should be studied profoundly. Furthermore, it will be necessary to develop a bi-layered PCL HF, in which graphene must be avoid in the luminal surface for HUVEC cells culture, and accounting with a PCL/G external layer for growing C6 cells with the objective of designing functional in vitro BBB models where 3D HUVEC/C6 co-cultures could be performed.

## 4. Conclusions

This work reports for the first time the fabrication of PCL/graphene hollow fibers by phase inversion to be used as tubular scaffolds for cell culture. In particular, the present work aimed to explore the viability of these novel PCL/G HFs to the future development of a dynamic in vitro BBB model. In order to accomplish this goal, the morphological, chemical, and functional properties of PCL and PCL/G HFs were characterized. The morphological analysis of the HFs found out that both types of membranes presented similar interconnected porosity (approx. 85%) but relevant differences in the average pore size. Particularly, the values obtained for PCL/G HFs (0.89 ± 0.08 µm) were significantly higher than the average pore size of polypropylene HFs used normally in dynamic in vitro BBB models (0.5 µm) and this provided high water fluxes sufficient to sustain neural models in vitro. Both PCL and PCL/G HFs mechanical properties were sufficient to enable their use as scaffolds for cell culture in BBB models.

As a preliminary evaluation of the potential of the herein reported HFs to develop in vitro BBB models, endothelial HUVEC and C6 cell lines were cultured individually and the adhesion, proliferation, and differentiation capacity on the surface of these HFs was analyzed. These studies showed that the higher electrical conductivity of PCL/G HFs could induce electroactive properties to the polymer membrane that resulted beneficial for C6 cells differentiation to astrocytes while graphene produced a cytotoxic effect on endothelial HUVEC cells. For the assembly of a functional in vitro BBB model, it is necessary to be able to produce 3D co-cultures of HUVEC/C6 in the same hollow fiber specimen. Therefore, our results highlight that it is necessary to develop a dual-layer HF that counts with the presence of graphene in the outer surface where C6 cells would be grown and its absence in the lumen for HUVEC cells culture. For that, co-extrusion techniques will be studied and applied.

## Figures and Tables

**Table 1:** Spinning conditions and process parameters for poly(ε-caprolactone) (PCL) and PCL/G hollow fiber (HF) synthesis.

Spinning Conditions	
Tube in orifice spinneret (mm)	OD: 1.3, ID: 0.7
Dope temperature (°C)	16
Dope flow rate (cm^3^ h^−1^)	150
Bore composition (wt%)	80/20 NMP/H_2_O
Bore flow rate (cm^3^ h^−1^)	64
Air gap height (cm)	1.5
Room temperature (°C)	18.8
Room humidity (%)	87.7
Quench bath composition (wt%)	10/90 NMP/H_2_O
Quench bath temperature (°C)	25
**Sequential Washing and Drying Procedure**	
(1) Water baths	1 (24 h)
(2) EtOH baths	3 (20 min each)
(3) Ambient dry	12 h

**Table 2:** Morphological characteristics of the PCL and PCL/G HFs. Student’s t-test statistical analysis of significance considering PCL HF as reference: OD, ID, and HF thickness (*n* ≥ 4), mean surface pore size, and bulk porosity (*n* = 3), * *p* < 0.05.

Parameter	PCL	PCL/G
Outer diameter, OD (µm)	1474 ± 22	1789 ± 43 (*)
Inner diameter, ID (µm)	908 ± 32	1080 ± 35
HFs thickness (µm)	273 ± 44	304 ± 74
Selective layer thickness, δ (µm) ^1^	1.41 ± 0.12	0.60 ± 0.04 (*)
Surface pore size (µm)	0.71 ± 0.04	0.89 ± 0.08 (*)
Bulk porosity, ε (%)	84.75 ± 0.84	83.56 ± 0.44

^1^ Estimated by using Hagen–Poiseuille porous model, Equation (4) and permeance values (see Section 3.2).

**Table 3:** Comparison between the mechanical parameters of the PCL and PCL/G HFs derived from the tensile stress-strain curves. Student’s t-test statistical analysis of significance considering PCL HF as reference, *n* = 5, * *p* < 0.05, ** *p* < 0.005.

Parameter	PCL	PCL/G
Young Modulus (MPa)	17.34 ± 0.79	16.62 ± 0.97
Yield point (MPa)	0.20 ± 0.03	0.24 ± 0.02 (*)
Ultimate tensile strength (MPa)	1.65 ± 0.03	1.40 ± 0.13 (**)
Elongation at break (%)	488.5 ± 46.4	327.1 ± 50.2 (**)

**Table 4:** Comparison of the hydraulic permeances of the present PCL and PCL/G HFs with other polymer membranes reported in the literature.

Membrane Material	Hydraulic Permeance (L m^−2^ h^−1^ bar^−1^)	References
PCL	33 ± 7	Present work
PCL/G	119 ± 13	Present work
PCL	238	Salerno et al. [16]
PAN	146	Morelli et al. [50]
PEEK-WC	30	Morelli et al. [51]
PLGA	20	Shearer et al. [52]
PLA	120	Moriya et al. [53]

## Supplementary Materials

The following are available online at https://www.mdpi.com/2077-0375/10/8/161/s1, Figure S1: Representative images of confocal microscopy for the study of astrocyte differentiation for 72 h of C6 cells grown on flat PCL membranes functionalized with either 0% graphene (A-B), 0.1wt% of GO (C-D) or 0.1 wt% of reduced GO (rGO) (E-F). Immunolabeling of GFAP (red channel) reveals astrocyte-differentiated cells, while F-Actin (green channel) counterstained all the cells in the field. (G) Quantification of percentage of GFAP-positive cells. Bars represent the mean ± SD. Student’s t-test statistical analysis of significance (*n* ≥ 4) were carried out considering ** *p* < 0.005, *** *p* < 0.0005. Scale bar: 100 μm.); Figure S2: Representative fluorescence images of PCL HF and PCL/G HF. DIC images were taken as positional reference of the HF. Green and red channels images were acquired using acquisition times 100–1000X higher (4000 msec) than regular epifluorescence images of stained cells cultured on HF.
