# Supplementary material for: Hollow Fiber Membranes of PCL and PCL/Graphene as Scaffolds with Potential to Develop In Vitro Blood—Brain Barrier Models
Source: Membranes (Basel). 2020 Jul 22;10(8):161. doi: 10.3390/membranes10080161 (PMC7464335; doi:10.3390/membranes10080161)
Supplement: Supplementary file 1 [file membranes-10-00161-s001.pdf]

Supplementary Materials

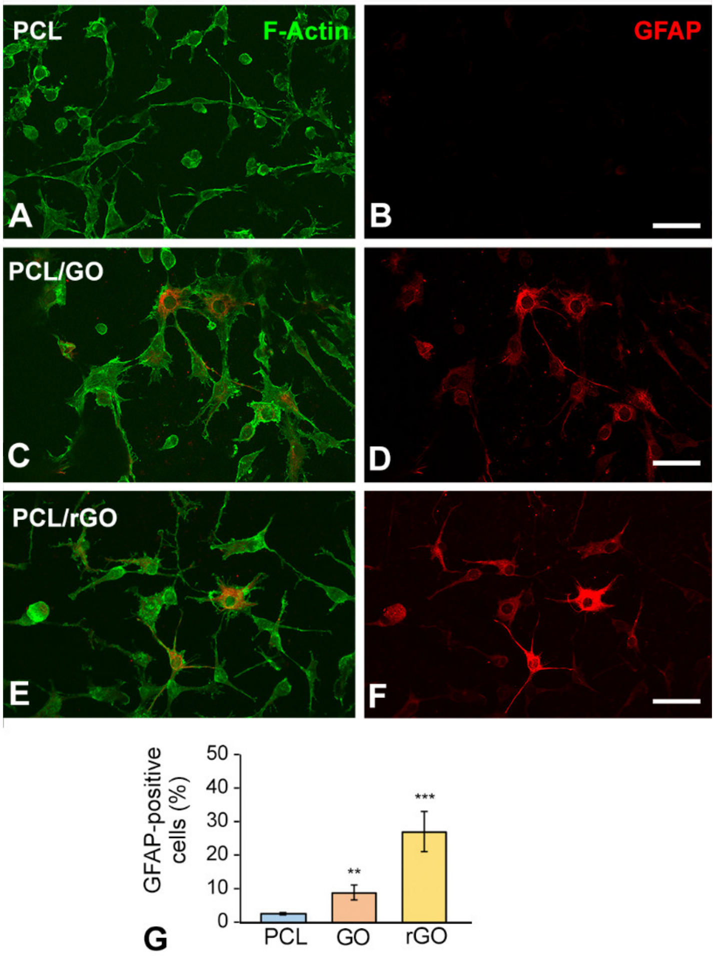

Figure S1. Representative images of confocal microscopy for the study of astrocyte differentiation for 72 hours of C6 cells grown on flat PCL membranes functionalized with either 0% graphene (A-B), 0.1wt% of GO (C-D) or 0.1 wt% of reduced GO (rGO) (E-F). Immunolabeling of GFAP (red channel) reveals astrocyte-differentiated cells, while F-Actin (green channel) counterstained all the cells in the field. (G) Quantification of percentage of GFAP-positive cells. Bars represent the mean ± SD. Student's t test statistical analysis of significance ($n \geq 4$) were carried out considering ** $p < 0.005$, *** $p < 0.0005$. Scale bar: 100 µm.

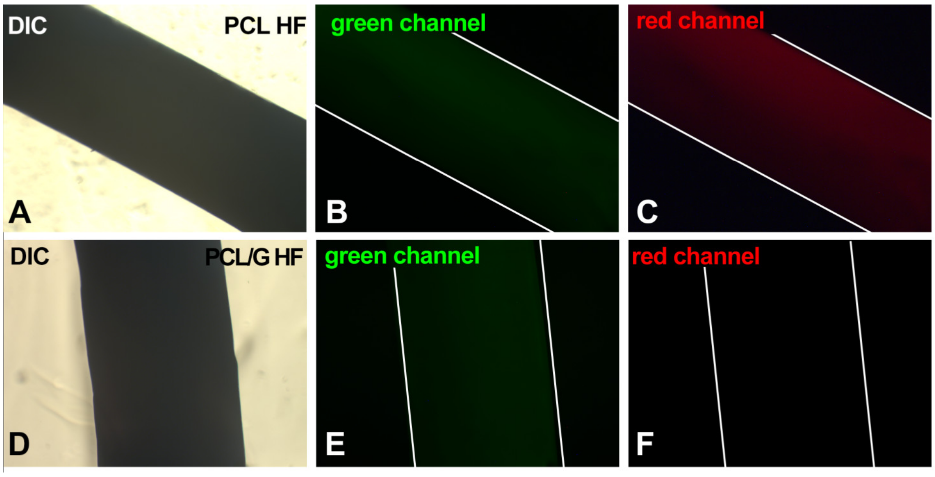

Figure S2. Representative fluorescence images of PCL HF and PCL/G HF. DIC images were taken as positional reference of the HF. Green and red channels images were acquired using acquisition times 100-1000X higher (4000msec) than regular epifluorescence images of stained cells cultured on HF.
